# Assessing real-world movements using consumer-grade wearable devices: Measuring segment orientations and movement quality

**DOI:** 10.1017/wtc.2025.10034

**Published:** 2025-11-19

**Authors:** T. Alexander Swain, Melitta A. McNarry, Samuel Manzano-Carrasco, Kelly A. Mackintosh

**Affiliations:** 1 Applied Sports, Technology, Exercise and Medicine Research Centre (A-STEM), Swansea University, Swansea, UK; 2 Medical School, Swansea University, Institute of Life Science 1, Swansea, UK; 3 Department of Communication and Education, Universidad Loyola Andalucía - Campus de Sevilla, Sevilla, Spain

**Keywords:** wearable technology, IMU, motion capture, motor skills, exercise, physical activity

## Abstract

In recent years, there has been growing interest regarding the impact of human movement quality on health. However, assessing movement quality outside of laboratories or clinics remains challenging. This study aimed to evaluate the capabilities of consumer-grade wearables to assess movement quality and to consider optimal sensor locations. Twenty-two participants wore Polar Verity Sense magnetic, angular rate, and gravity (MARG) sensors on their chest and both wrists, thighs, and ankles, while performing repeated bodyweight movements. The Madgwick sensor-fusion algorithm was utilized to obtain three-dimensional orientations. Concurrent validity, quantified using the root-mean-square-error (RMSE), was established against a Vicon optical motion capture system following time-synchronization and coordinate-system alignment. The chest sensors demonstrated the highest accuracies overall, with mean RMSE ( 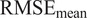 ) less than 9.0° across all movements. In contrast, the wrist sensors varied considerably ( 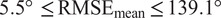 ). Ankle and thigh sensors yielded mixed results, with the 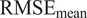 ranging from 2.0° to 40.0°. Notably, yaw angles consistently demonstrated higher discrepancies overall, while pitch and roll were relatively more stable. This study highlights the potential of consumer-grade MARG sensors to increase the real-world applicability and accessibility of complex biomechanical models. It also accentuates the requirement for strategic sensor placement and refined calibration and postprocessing methods to ensure accuracy.

## 1. Introduction

Following the technological revolution, wearable technology has become an integral part of modern lives. Current wearable devices offer an array of functionalities, including the capability to provide numerous health-related metrics (Zhang et al., 2019). However, while wearables are increasingly used to promote physical activity and exercise among the general population, such devices have primarily focused on the quantification of movement (Venek et al., 2022). Yet, there is growing awareness of the concomitant importance of movement quality as well as movement quantity, although this remains an often-overlooked component of physical activity (Rudd et al., 2020). Indeed, quality movement is vital for minimizing injury risks, while also enabling enhanced athletic performance (Venek et al., 2022). Moreover, life expectancy may also be extended by increasing individuals’ motivation and confidence to engage in physical activity (Robinson et al., 2015).

Historically, movement quality has been measured and evaluated within research and clinical settings. In clinical environments, assessments are typically conducted subjectively by movement experts, with optical motion capture (OMC) commonly utilized as the gold standard in research-based evaluations (O’Reilly et al., 2018). However, assessing movement quality outside of these constraints remains challenging, due to substantial time, financial, and spatial limitations (Adesida et al., 2019; Kruk and Reijne, 2018). Moreover, the applicability of these environments and perhaps the movements typically analyzed may constrain ecological validity (Skjaerven et al., 2015Kruk and Reijne, 2018; Anwary et al., 2021). Consequently, the wider population are generally precluded from the benefits associated with understanding and enhancing their movement quality. Wearable technology presents a potential opportunity to overcome these restrictions by offering an affordable, accessible, and practical method of assessing movement quality (O’Reilly et al., 2018).

Prior research has shown that wearable technology, predominantly magnetic, angular rate, and gravity (MARG) sensors, inertial measurement units (IMUs), or their constituent components, can be implemented in the assessment of movement quality by directly measuring and quantifying motion characteristics (Swain et al., 2023). Often, this centers around the computation of sensor orientations, which, consequently, also permits the estimation of joint angles (Swain et al., 2023). However, despite extensive research validating the capabilities of wearables to measure orientations during physical activity (Shepherd et al., 2017; Mitternacht et al., 2022; Shuai et al., 2022), such validation studies are not without limitations. IMUs and MARG sensors, for example, are frequently validated against OMC systems during physical activity (Swain et al., 2023). In such validations, sensors are typically secured to the superficial rigid reflective marker clusters that are commonly used in motion capture systems, ensuring the orientation of the cluster directly corresponds to that of the device body (Beange et al., 2019; Teufl et al., 2019; Lin et al., 2021; Michaud et al., 2021). However, while this approach enables precise validation of the sensor orientation data, it fails to address the nuances of movement quality; the orientation-tracking capabilities of the sensors are validated without consideration of movement standards or the dynamic, interconnected movements of the human body and its skeletal structure. Therefore, a lack of practical transferability becomes evident, as OMC systems utilize skeletal-based biomechanical models to assess human movement, with motion described relative to joint centers rather than the superficial marker placements.

The current literature, while demonstrating the potential for wearable technology to aid the assessment of movement quality, is largely dominated by uniplanar and single-joint movements (Beange et al., 2019; Meng et al., 2019; Lin et al., 2021). Such studies, which predominantly utilize clinical- or research-grade sensors (Tulipani et al., 2018; Cortesi et al., 2019; Michaud et al., 2021), do not fully capture the complex, multidimensional nature of human motion. Additionally, there is a paucity of studies validating multiple triaxial MARG sensor orientations for full-body compound movements relative to standardized biomechanical models. Indeed, only two studies have sought to validate the orientations of wearable devices relative to such models, both of which used research-grade devices to examine primarily lower-limb movements for clinical applications (Dahl et al., 2020; Niswander et al., 2020). Moreover, although research has shown that sensors can accurately quantify physical activity irrespective of placement (Mackintosh et al., 2016), other studies suggest their performance may decline during movement quality assessments if the sensor positioning is suboptimal (Swain et al., 2023). Therefore, this study aimed to evaluate the capability of consumer-grade wearables in assessing movement quality during compound and functional exercises in comparison to established OMC models, encompassing both the upper and lower body. As a secondary aim, the study sought to identify the influence of sensor location on orientation measurements for use in movement quality assessments, given the potential impact this may have on sensor accuracy.

## 2. Methods

### 2.1. Data collection

A total of 24 healthy adult participants (aged over 18 years) volunteered for the study. Two participants were excluded from the analysis: one due to data corruption, and the other due to a technical error. The remaining 22 participants (17 male) had a mean age, body mass, and height of 28.5 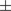 7.1 years, 74.1 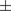 13.8 kg, and 172 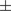 9 cm, respectively.

Before participation, individuals provided written informed consent and completed a Physical Activity Readiness-Questionnaire (PAR-Q; Warburton et al., 2019). Thereafter, health screening was also conducted for each participant in accordance with the American College of Sports Medicine (ACSM) Risk Stratification Chart (Sports Medicine, 2019). This study received ethics approval from the Swansea University Faculty of Science & Engineering Research Ethics & Governance Sub-Committee (approval ref: 1 2023 6331 5423).

**Figure 1.**
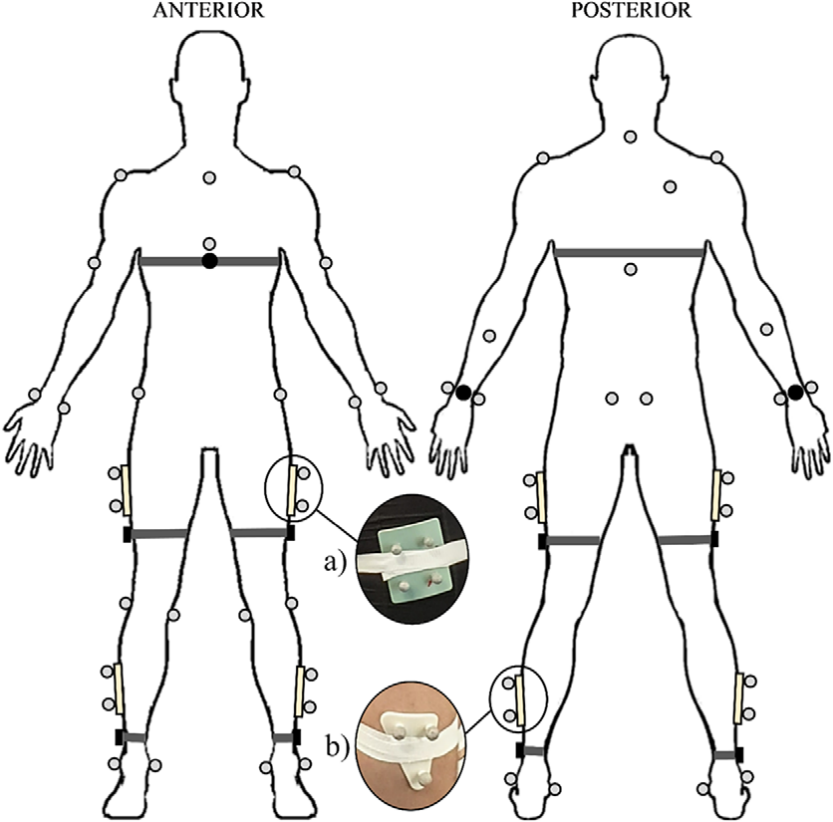
Indicative placement of Polar Verity Sense devices and Vicon optical motion capture system reflective markers utilized in this study. The inserts show the marker clusters used on the (a) lateral thighs and (b) lateral shanks.

Each participant wore eight bluetooth-equipped Polar Verity Sense 9-axis MARG sensors (Polar Electro Oy, Oulu, Finland), each featuring an accelerometer ( 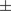 8 g, 52 Hz), gyroscope ( 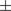 2,000 dps, 52 Hz), and magnetometer (50 G, 100 Hz). The devices utilized in this study were placed on the chest over the xiphoid process, the dorsal side of each wrist at the distal end of the radius, above the lateral malleolus on each ankle, and laterally on the thighs approximately halfway between the greater trochanter and lateral epicondyle (Figure 1). Participants also wore a device positioned at the lumbosacral region of the lower back. However, data from this device were not included in the present analysis. The sensors were remotely operated via the Polar Sensor Logger V17 Android application (J. Happonen, Kempele, Finland). Elasticated fabric straps or adhesive tape were used to secure the devices. Concurrently, kinematic motion data were captured at a sampling rate of 250 Hz using a 12-camera Vicon OMC system and Nexus 2.0 software (Vicon Motion Systems Ltd., Oxford, UK). A standardized and widely utilized biomechanical model and reflective marker set, Plug-In Gait (PiG; Vicon Motion Systems Ltd., Oxford, UK; Baker et al., 2018; Leboeuf et al., 2019; Samala et al., 2020), was used as the gold standard measure (Figure 1), with marker clusters used on the lateral surfaces of participants’ shanks and thighs to enhance measurement accuracy (Mackintosh et al., 2016; Sports Medicine, 2019; Warburton et al., 2019; Dahl et al., 2020; Samala et al., 2020). Furthermore, based on the sensor placements, the PiG head and feet markers, and corresponding segments, were excluded from the analysis, as they were extraneous to the study’s focus.

**Figure 2.**
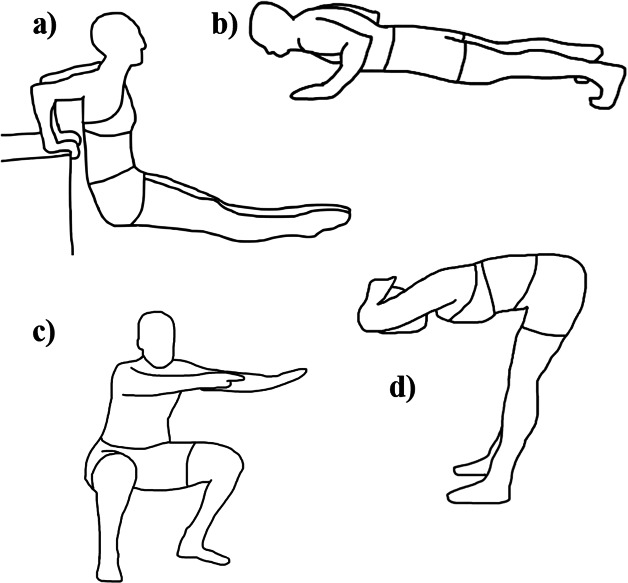
Exercises performed during data collection (a) chair dip, (b) push-up, (c) squat, and (d) good morning.

Participants performed a warm-up consisting of 5 minutes on a Concept2 Model E rowing ergometer (Concept2 Inc., Morrisville, VT, USA) followed by a range of dynamic stretches. Each participant was then asked to complete a protocol involving five exercises: squats, push-ups, good mornings, chair dips, and ab crunches. Ab crunch data and the chest-sensor data for push-ups were subsequently excluded from the analysis due to positional OMC marker occlusion. The four movements included in the analysis are shown in Figure 2. All participants deemed themselves physically capable of performing the prescribed movements. Following a brief demonstration, each movement was performed for three sets of 10 repetitions, with each set and each movement interspersed with a 2-minute period of passive recovery. The exercises were randomized within a stratified order of: upper, lower, upper, lower, upper, where the squat and good morning were considered to be lower body exercises and the push-up, chair dip, and ab crunch were upper body movements. The Vicon OMC system and Polar Verity Sense devices were independently triggered prior to each set to simultaneously capture movement data and were subsequently terminated once each set had concluded. Before commencement and following completion of each set of the prescribed exercises, participants were asked to maintain a 5-second static pose to establish a baseline (Tulipani et al., 2018).

### 2.2. Data processing and analysis

**Figure 3.**
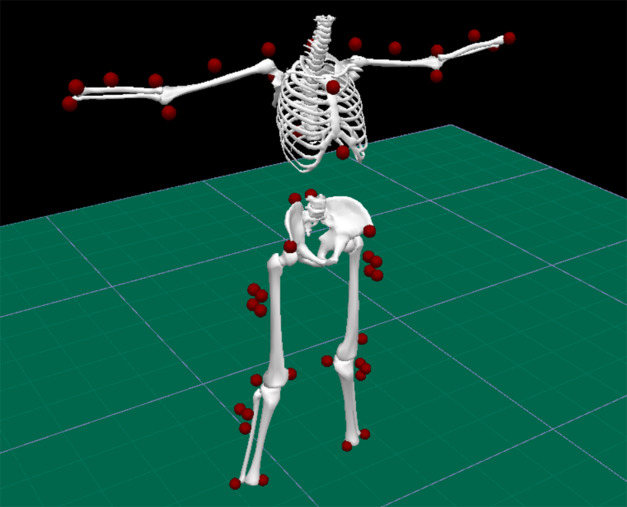
Visual3D biomechanical model developed from the modified Plug-In Gait marker set.

On conclusion of the data capture, where required, the Vicon recordings underwent gap filling to overcome marker occlusion. Thereafter, the C3D marker coordinate files generated by Vicon were exported to Visual 3D (C-Motion Inc., Germantown, MD, USA) biomechanical modeling and analysis software program. A biomechanical model was constructed using the modified PiG marker set, comprising 10 segments: thorax, upper arms, lower arms, pelvis, thighs, and shanks (Figure 3). Orientations for each limb segment were then extracted, with each segment’s local coordinate frame approximately aligned to that of the corresponding sensor.

**Figure 4.**
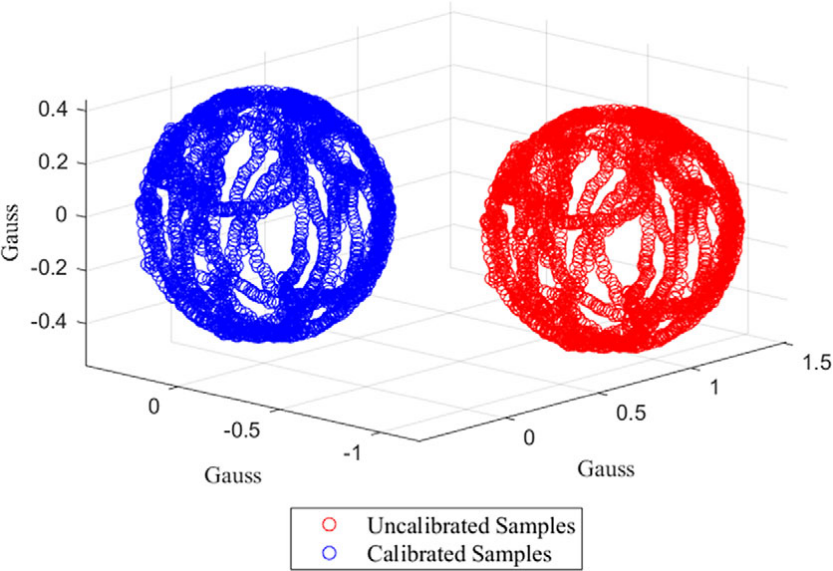
Magnetometer calibration process with raw precalibrated data in red and postcalibration data in blue. The shift in distribution following calibration should be noted with the postcalibration data (blue) centered.

The data for each constituent component of the MARG sensors were stored locally as .txt files on the Android device used for data capture and were subsequently transferred to MATLAB R2023a (The MathWorks Inc., Natick, MA, USA) for analysis. To correspond with the OMC segment frame of reference, the sensor data were transformed from a left- to right-handed coordinate system. Next, the magnetometer and gyroscope components were retrospectively calibrated; the MATLAB function “magcal” was utilized for the magnetometer (Figure 4; Magnetometer calibration coefficients – MATLAB magcal – MathWorks United Kingdom, n.d.; Ozyagcilar, 2015), whereas a proprietary function was used for the gyroscope. Notably, the sensor data were subject to filtering during data capture utilizing a proprietary “black box” algorithm. Hence, no additional filtering was required; this was confirmed via frequency analysis. The magnetometer data, initially sampled at 100 Hz, were downsampled to 52 Hz to correspond with that of the accelerometer and gyroscope, ensuring uniform sampling rates across all sensors. Moreover, while each sensory component within the MARG sensor recorded data simultaneously, the start times were asynchronous due to varying initialization durations for each component. To synchronize, the MARG sensor data were cropped to ensure a common temporal origin while isolating the relevant data segments exclusively. The Madgwick gradient descent sensor fusion algorithm was then applied to the individual datasets obtained from the MARG sensor components to estimate the orientation of the device, utilizing the default 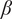 -value of 0.1 (Madgwick et al., 2011; Open source IMU and AHRS algorithms – x-io Technologies, n.d.). Orientations were expressed using classical Euler parameters in the XYZ sequence, representing pitch, roll, and yaw rotations, respectively.

The OMC orientation data, also utilizing the XYZ convention, were imported into MATLAB for comparison with the processed sensor data. To facilitate this, the sensor orientation data underwent resampling to align its sampling rate with that of the OMC data. An essential step in the analysis was the alignment of the global coordinate systems for both the OMC and MARG sensors. The OMC global coordinate system, established during system calibration, served as the laboratory central reference point. In contrast, the Madgwick sensor fusion algorithm, influenced by the magnetometer, outputs orientations in a geomagnetic coordinate system, defined by the heading relative to magnetic north (Kok et al., 2017). Consequently, a misalignment exists that predominantly affects the measurement of rotation about the *z*-axis (i.e., the yaw angle; de Vries et al., 2009). To rectify this, the yaw angle discrepancies between the OMC segments and corresponding MARG sensors were determined under static conditions, utilizing the mean yaw angles to compensate for signal noise. Thereafter, the Direction Cosine Matrix (DCM) was calculated for each sensor orientation data point along the temporal sequence. A rotation matrix, based on the yaw angle discrepancy, was subsequently applied to each DCM to align the coordinate systems, with the adjusted DCMs utilized to derive updated Euler angles (Niswander et al., 2020).

**Figure 5.**
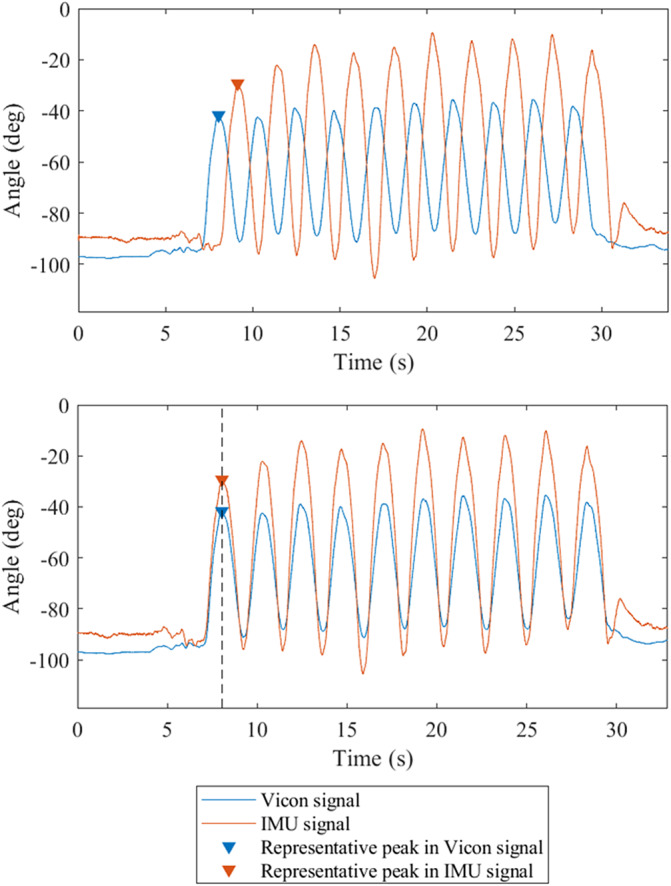
Representative data showing thorax orientations. The upper panel shows the orientation signals prior to synchronization, illustrating the example signal features before alignment. The lower panel shows the signals following synchronization, demonstrating alignment of the peaks.

Due to the independent recording of each system, synchronization of the OMC and MARG sensors was enabled by utilizing distinct signal features within the orientation outputs (Figure 5; Shepherd et al., 2017; Beange et al., 2019; Cortesi et al., 2019). Next, the data were baselined to zero to facilitate the comparison of the absolute orientations.

To assess concurrent validity, the orientation discrepancies between the OMC and Polar Verity Sense systems were quantified using root-mean-square-error (RMSE), frequently employed in similar studies for evaluating waveforms (Tulipani et al., 2018; Lin et al., 2021; Michaud et al., 2021; Mitternacht et al., 2022; Shuai et al., 2022). The mean RMSE (RMSE_mean_) for the decoupled Euler parameters across all participants was calculated for each sensor location and each movement (Table 1). Outliers were identified and excluded using a *z*-score confidence interval of 95% (*z* = 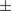 1.96). To categorize the efficacy of the sensors, the thresholds recommended by Poitras et al. (2019) were utilized; RMSE values below 5° were deemed “excellent”, those ranging between 5° and 10° were classified as “good”, and any RMSE values exceeding 10° were considered “unacceptable”.

**Table 1.** RMSE_mean_ (SD) for sensor placements during different movements (°)

Movement	Euler parameter	RMSE_mean_ (SD) for sensor placements (°)						
		C	LA	LT	LW	RA	RW	RT
Squat	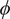	6.3 (2.8)	5.1 (2.4)	10.1 (3.9)	9.3 (7.2)	6.3 (4.2)	7.3 (4.6)	21.7 (25.1)
	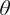	2.8 (1.8)	7.1 (4.7)	29.9 (14.7)	9.0 (11.1)	6.5 (3.7)	16.7 (8.6)	10.4 (10.7)
	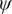	8.3 (3.0)	27.2 (21.9)	37.5 (17.5)	17.3 (10.4)	13.3 (11.7)	19.6 (12.1)	30.1 (19.8)
GM	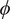	7.1 (3.0)	2.6 (1.3)	5.9 (2.7)	90.3 (62.0)	2.4 (1.2)	4.7 (1.9)	74.9 (39.4)
	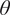	2.7 (1.0)	2.0 (0.9)	10.7 (8.0)	42.5 (20.2)	7.5 (5.2)	11.9 (6.9)	37.9 (12.9)
	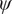	8.9 (4.1)	6.0 (3.4)	5.7 (2.7)	84.3 (66.5)	2.4 (1.6)	3.4 (1.9)	139.1 (54.5)
PU	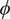	–	22.5 (19.0)	17.8 (12.9)	22.2 (20.5)	18.7 (13.6)	32.5 (40.4)	9.1 (4.4)
	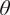	–	8.8 (2.7)	4.5 (2.2)	11.2 (6.4)	6.7 (3.5)	3.9 (1.9)	7.7 (6.1)
	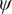	–	50.6 (31.8)	33.8 (20.2)	42.9 (34.2)	66.1 (43.6)	54.6 (40.3)	21.0 (11.9)
CD	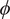	7.7 (3.6)	40.0 (38.7)	27.4 (19.8)	8.1 (7.3)	39.0 (29.8)	35.9 (34.8)	5.5 (3.0)
	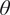	2.2 (1.0)	9.4 (11.9)	9.5 (7.5)	12.5 (6.9)	9.5 (10.0)	10.2 (7.4)	9.8 (5.6)
	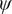	2.4 (1.0)	45.9 (36.9)	35.1 (30.1)	58.6 (53.9)	47.5 (34.6)	39.7 (42.0)	16.2 (9.4)

*Note:* 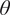 roll, 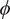 pitch, *ψ* yaw. Utilizing the thresholds recommended by Poitras et al. (2019), “excellent” RMSE values (<5*°*) are highlighted in green, “good” RMSE values (5–10*°*) are uncolored, and “unacceptable” RMSE values >10*°* are shown in red.

Abbreviations: C, chest; CD, chair dip; GM, good morning; LA, left ankle; LT, left thigh; LW, left wrist; PU, push-up; RA, right ankle; RMSE_mean_, mean root-mean-square-error; RT, right thigh; RW, right wrist; SD, standard deviation.

## 3. Results

**Figure 6.**
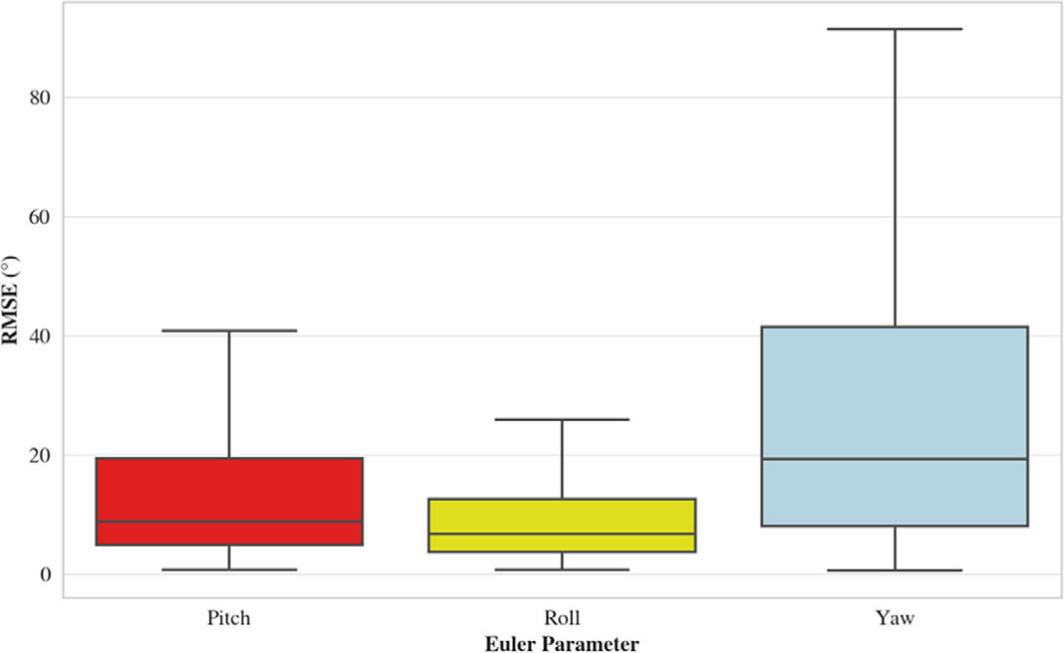
Box plots for RMSE based on Euler parameters.

The overall RMSE values for the decoupled Euler parameters indicate that the yaw-angle estimates were most erroneous across all movements and sensor locations (Table 1 and Figure 6; for specific movement and sensor locations, please see Supplementary Material 1). Only the chest sensor was consistently accurate in the yaw direction, along with the left ankle and thigh sensors for the good morning exercise. The greatest yaw angle errors were produced by the wrist-worn sensors.

The roll-angle measurements typically yielded the best results. For the squat exercise, chest sensors were consistently accurate (Table 1). Conversely, sensors placed on the left thigh and right wrist exhibited inaccuracies for the pitch and roll angles, in addition to the right thigh sensor in the roll direction, exclusively. All other pitch and roll measurements for the squat were within the tolerable range (Poitras et al., 2019). During the good morning exercise, the accuracy of pitch angles varied considerably; the chest, thigh, and ankle sensors ranged from “good” to “excellent”, although the wrist sensors generated substantial inaccuracies. Similarly, the wrist-sensor estimates were highly erroneous in the roll direction, with inaccuracies also observed for the thighs. However, the chest and ankle measurements were within the reasonable accuracy limits. For push-ups, the right wrist sensor uniquely exhibited tolerable pitch-angle accuracy, contrasting with the higher errors of other placements. In the roll direction, only the left wrist sensor surpassed the acceptable limit; the thigh sensors notably displayed “excellent” accuracies. For chair dips, the chest sensor accuracies were within the tolerable accuracy range in all directions. The wrist sensors performed adequately in the pitch direction, with less reliable estimates obtained for the ankle and thigh sensors. For the roll angles, unacceptable errors were observed for the left wrist and right thigh, although all other sensor positions returned “good” to “excellent” accuracies.

## 4. Discussion

The results of this study suggest that the accuracy of orientation measurements using MARG sensors is movement specific and may also be impacted by anatomical sensor positioning. Further, it highlights the challenges associated with using superficially positioned wearables to mimic established OMC skeletal models, the gold-standard for movement quality assessments (Samala et al., 2020).

Previous research has suggested flexibility regarding sensor placement when measuring movement quantity. Mackintosh et al. (2016), for example, found no significant difference in energy-expenditure measurements across a broad range of anatomical sensor placements, or with the use of multiple devices, thereby offering consumers the advantage of choosing their preferred sensor placement. Unsurprisingly, research has demonstrated that compliance is best achieved using a wearable device worn on the wrist (Trost et al., 2014). However, it may not be feasible to capitalize on the ubiquity of wrist-worn devices for the assessment of movement quality. Notably, the current study highlights that the commonality of sensor positions for quantifying energy expenditure does not translate to movement quality assessments, with key distinctions evident according to sensor location and movement type.

Chest-worn devices were the most consistently accurate across all exercises, likely aided by the minimization of soft tissue artefact error due to the bony sternal placement. This aligns with the findings of Dahl et al. (2020), who evaluated research-grade sensors for similar movements and sensor placements using a comparable OMC biomechanical model. This finding is significant, given the value of sensors positioned on the torso for the assessment of movement quality. Speculatively, torso-worn sensors may offer transferability across numerous activities due to the proximity to the body’s center of mass, congruent with observations derived from machine learning activity recognition (Rahmani et al., 2021) and movement discrepancy detection (O’Reilly et al., 2017a, 2017b, 2017c). Indeed, while multiple devices typically offer more holistic capabilities than single units (Swain et al., 2023), evidence shows that coarse insights can be made regarding the quality of compound movements with single units worn on the torso, particularly for lower body dominant activities, such as walking (Caporaso and Grazioso, 2020), squatting (O’Reilly et al., 2017c), and lunging (O’Reilly et al., 2017b). Further, torso-worn sensors, typically positioned on the chest or back, have been effectively utilized to measure the kinematics of hip hinge (Michaud et al., 2021) and spinal motions (Beange et al., 2019; Brouwer et al., 2021; Michaud et al., 2021), essential components of a diverse range of movements. Moreover, while not perceived to be as comfortable as some other anatomical positions, such as the wrist or hip, evidence indicates that chest-worn sensors are positively perceived overall (Beeler et al., 2018), and may therefore be viable for consumer-friendly movement quality assessments.

Accuracies observed at other placements in the current study were considerably more varied depending on the movement performed. A challenge with Euler-based orientation estimates derived from IMUs or MARG sensors is gimbal lock, where two of the three rotational axes of the gyroscope align, leading to the loss of one degree of freedom (Renaudin and Combettes, 2014). In the current study, this phenomenon was manifest as the rotation in the roll direction approached 90°, resulting in unpredictable orientation behavior in the pitch and yaw outputs. Alternative approaches, such as the use of quaternions, can be used to address this gimbal lock problem (Challis, 2020). Nonetheless, as most people are unfamiliar with quaternions, Euler angles remain more comprehensible (Mourcou et al., 2015). Gimbal lock was especially evident for the thigh and ankle sensors during chair dips and push-ups – the greater RMSE values in the pitch and yaw directions, with comparatively low RMSE values in the roll direction, strongly indicate the presence of gimbal lock, despite the limited motion actually experienced by the sensors. Furthermore, the wrist sensors exhibited similar characteristics during the good morning exercise, though with even greater error.

It is reasonable to speculate that the relatively high rotational velocity of the wrist sensors engendered during the movements involved in the current study contributed to the large errors, congruent with the effects observed in previous research (Lebel et al., 2013; Dahl et al., 2020), while the error was compounded by gimbal lock. These findings underscore the sensitivity of movement quality assessments to sensor placement, emphasizing the importance of both the body segment and the specific location on the segment that the devices are worn. Moreover, they reveal the current limitations of consumer-grade IMUs and MARG sensors in accurately measuring orientations for high-speed movements. This is in accord with Lebel et al. (2013), who highlighted the challenges posed by faster, more dynamic activities, suggesting that wrist-worn devices may consequently be unable to accurately assess the movement quality of everyday tasks.

Inaccuracies were prominent in the thigh and wrist sensors during the squat. It is postulated that, in alignment with prior research (Blandeau et al., 2023), the observed error from the thigh sensors was attributable to the influence of the upper leg musculature; underlying muscular contractions could engender measurement errors not experienced by a more rigid segment. It is also feasible that the presence of gimbal lock may have influenced some of the thigh sensor data during the squat. However, this would only arise where sufficient depth was achieved (i.e., thigh segments parallel to the floor; Kritz et al., 2009), which, in accordance with the OMC data, was only observed to be the case for a minority of participants (n = 3). For the wrist sensors, the outputs appear to be influenced by participant arm motion during the squatting movement. Many individuals incorporated an arm swing instead of maintaining a fixed arm position, with the associated heterogeneity complicating the identification of a clear overarching source of error. However, it is likely that the rapid and multidirectional nature of these arm swings contributed to the measurement inaccuracies.

The sensor measurements demonstrated good accuracy under static conditions, or indeed where range of motion was small. For example, the thighs and ankles during good mornings typically exhibited good accuracies as movement was minimal, while the ankle sensors performed well during squats in the pitch and roll directions, recognizing that the primary direction of motion was about the *z*-axis (i.e., yaw). However, the practical applications of static orientation measurements are limited when assessing human movement quality. Contrastingly, the wrist measurements during push-ups and chair dips, which remained mostly static, exhibited unexpected errors. For both exercises, the proximity of the sensors to the wrist joint during flexion may have contributed to the inaccuracies, indicating that a position further up the forearm may be more appropriate. However, it is also pertinent to note that distinct measurement discrepancies were observed between the left and right wrists, particularly during push-ups. While the exact sources of these errors remain unidentified, potential magnetometer interference could be a contributing factor.

When considering the decoupled Euler parameters in isolation, measurements in the yaw direction are considerably more erroneous than the pitch and roll. This is comparable with the findings of Bergamini et al. (2014), who explored an array of orientation estimation algorithms for measuring human movement, including the Madgwick algorithm (Madgwick et al., 2011). The errors were more pronounced during prolonged data capture, specifically during locomotion tasks, as compared to shorter, single-event manual tasks (Bergamini et al., 2014). This occurred despite the authors utilizing a method where research-grade sensors were securely affixed to rigid OMC marker clusters. The results presented by Bergamini et al. (2014) are analogous to the findings of this study, where data capture was continuous across multiple repetitions. Building on these observations, the findings of the current study suggest that the pitch estimates were, overall, more erroneous than the roll angles. This is contrary to previous research, where pitch-angle estimates are typically most accurate, or indeed comparable, to the roll estimates (Kok et al., 2017; Madgwick et al., 2011), However, it is important to note that previous findings are typically from highly controlled studies, where the Euler parameters are tested under uniform conditions. The sensor placements and specific movements considered within this study introduced considerable variability. For example, the movements considered predominantly utilized the *x*- and *z*-axes as the primary rotational axes, with the *y*-axis acting as a secondary rotational axis. Moreover, gimbal lock influenced the error in pitch and yaw angle measurements exclusively, leaving the roll angle unaffected.

While this study presents valuable insights into the use of commercial-grade MARG sensors for measuring human movement, it is important to consider the findings in the context of certain limitations. The sensors utilized were manually aligned based on approximated positions relative to the local coordinates of the OMC system. This may have influenced the precision of the measurements, although this was accounted for where possible by considering absolute rather than relative orientations. Additionally, the reliance on magnetometers to correct gyroscope drift, while common, brings challenges such as environmental interference (Ligorio et al., 2020). Despite attempts to mitigate this issue, it is not possible to guarantee complete elimination of interference due to the nature of the movements performed, though this also highlights the real-world limitations of current sensor technologies.

Future work should aim to address magnetometer interference by applying corrective methods to improve sensor accuracy. Notably, the sensors require further validation under controlled conditions to better understand the observations of the current study, including the influence of movement speeds and range of motion. It is also suggested that gyroscope-based yaw-angle estimates could be reconsidered, noting that research has offered novel methods to counteract gyroscope integration drift, potentially eliminating the need for magnetometers (Han et al., 2018; Ligorio et al., 2020), notorious for their sensitivity to interference (Kok et al., 2017) and computational inefficiencies (Han et al., 2018). Following this, the increased prevalence of machine-learning methods in wearable technology applications offers an alternative avenue for the measurement and evaluation of movement quality (Kianifar et al., 2017; O’Reilly et al., 2017a, 2017b, 2017c; Spilz and Munz 2022). Machine-learning models can potentially offer greater understanding of complex movements by identifying patterns in the data and, in the context of human movement, machine-learning classification techniques could bypass some of the existing challenges associated with sensor kinematic measurement inaccuracies. Therefore, machine learning may offer an interim, or integrated, solution that can complement, or potentially replace, the use of sensor-derived kinematics and such methods warrant future consideration.

## 5. Conclusions

Overall, this study highlights the potential, but also inherent limitations, of using commercial-grade MARG sensors to assess human movement. While there is notable promise, particularly in the measurement of uniplanar motion and the use of chest-worn devices, practitioners must exercise caution when interpreting sensor-derived orientation data. This study provides a foundation for subsequent research aiming to refine these tools and enhance their accuracy in comparison to traditional biomechanical models for use in real-world applications, while highlighting machine-learning classification techniques as an alternative approach to assessing movement quality. The accessibility of wearable technology offers promising opportunities for the general public to gain insights into their movement quality, while contributing to a more comprehensive framework for physical activity and wellness monitoring.

## Supporting information

Swain et al. supplementary material 1 Swain et al. supplementary material

Swain et al. supplementary material 2 Swain et al. supplementary material

## Data Availability

Data can be made available to interested researchers upon request by email to the corresponding author.

## Supplementary material

The supplementary material for this article can be found at http://doi.org/10.1017/wtc.2025.10034.
