# Supplementary material for: Assessing real-world movements using consumer-grade wearable devices: Measuring segment orientations and movement quality
Source: Wearable Technol. 2025 Nov 19;6:e54. doi: 10.1017/wtc.2025.10034 (PMC12641298; doi:10.1017/wtc.2025.10034)
Supplement: Swain et al. supplementary material 2 — Swain et al. supplementary material [file S2631717625100340sup002.doc]

**Supplementary Material 1**

**Squats**


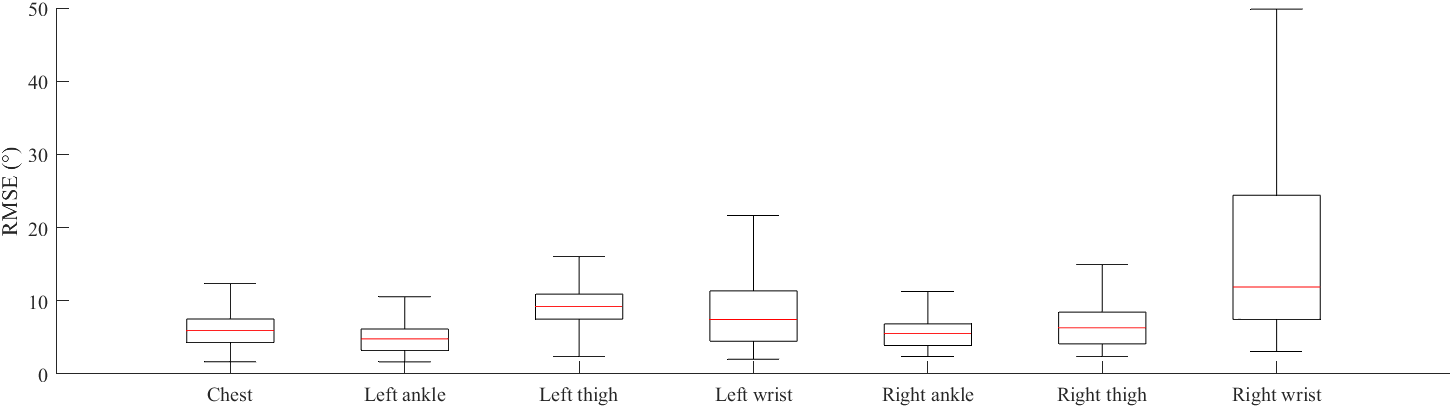


**Figure S1** Squat pitch angle box plots


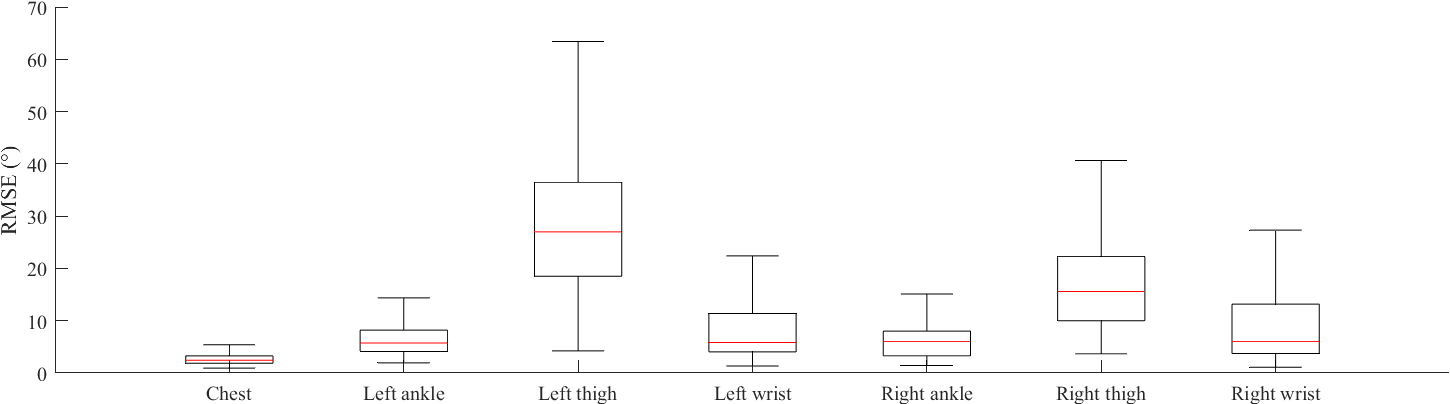


**Figure S2** Squat roll angle box plots


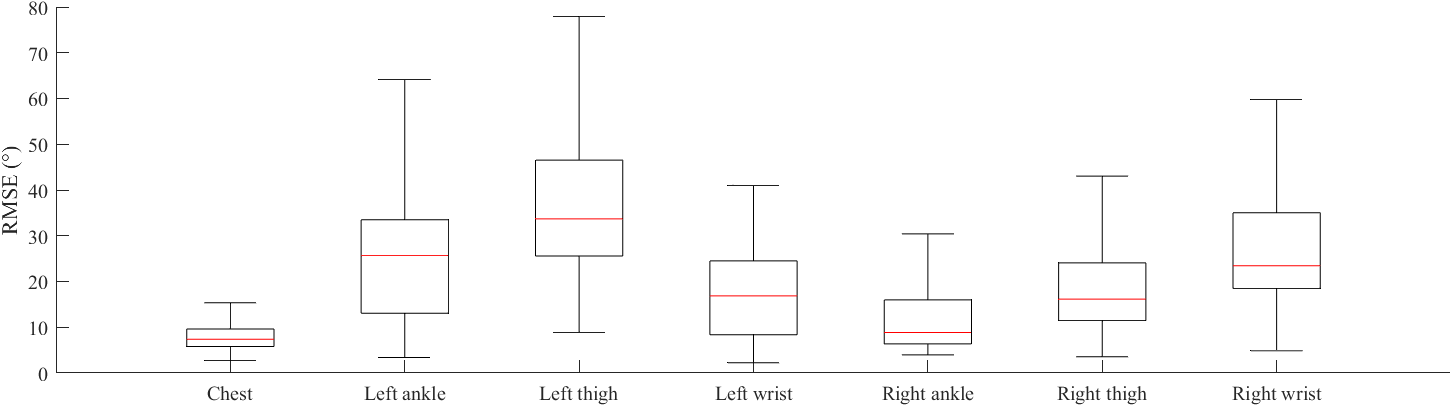


**Figure S3** Squat yaw angle box plots

**Good mornings**


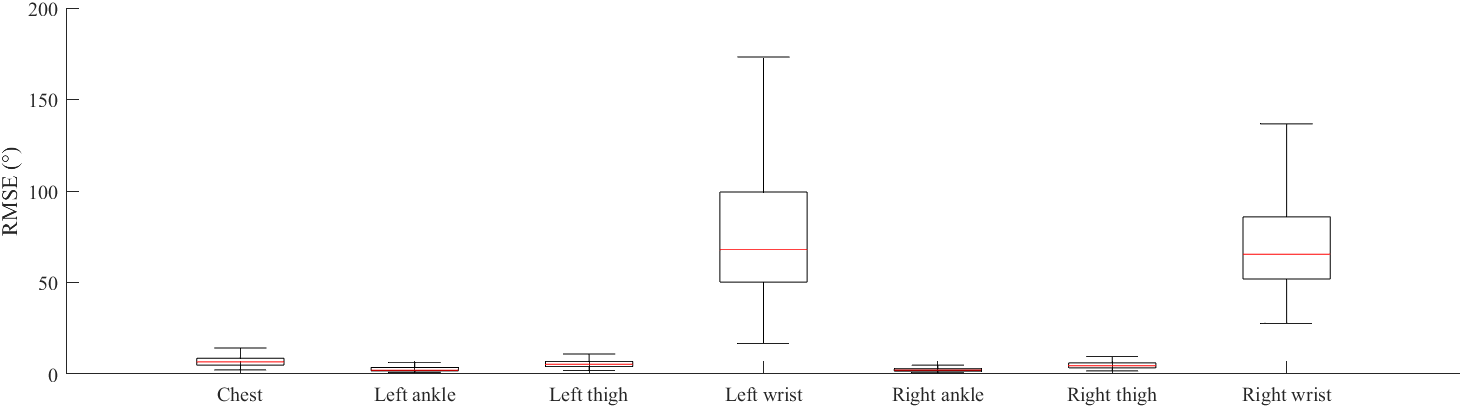


**Figure S4** Good morning pitch angle box plots


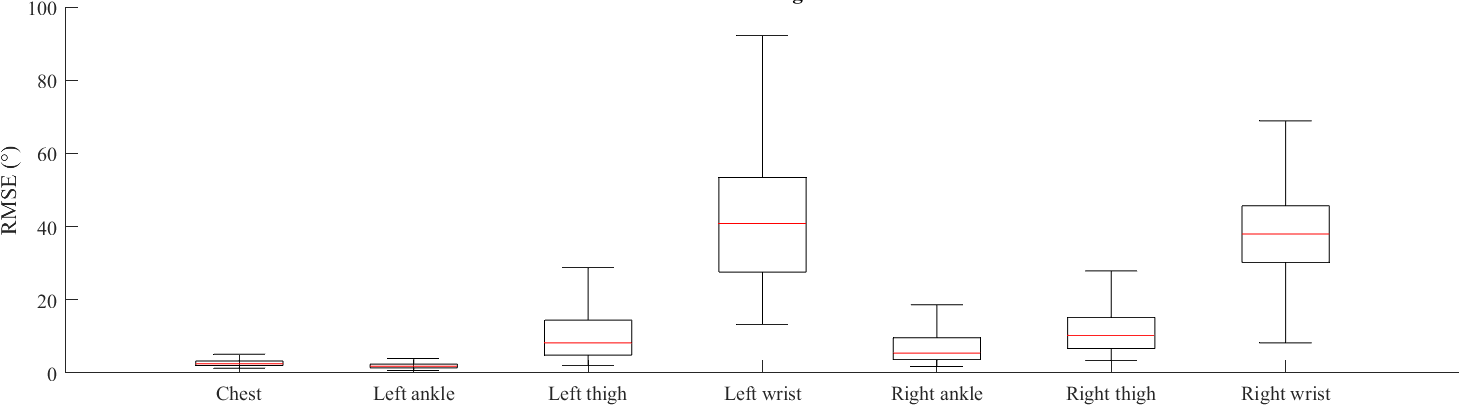


**Figure S5** Good morning roll angle box plots


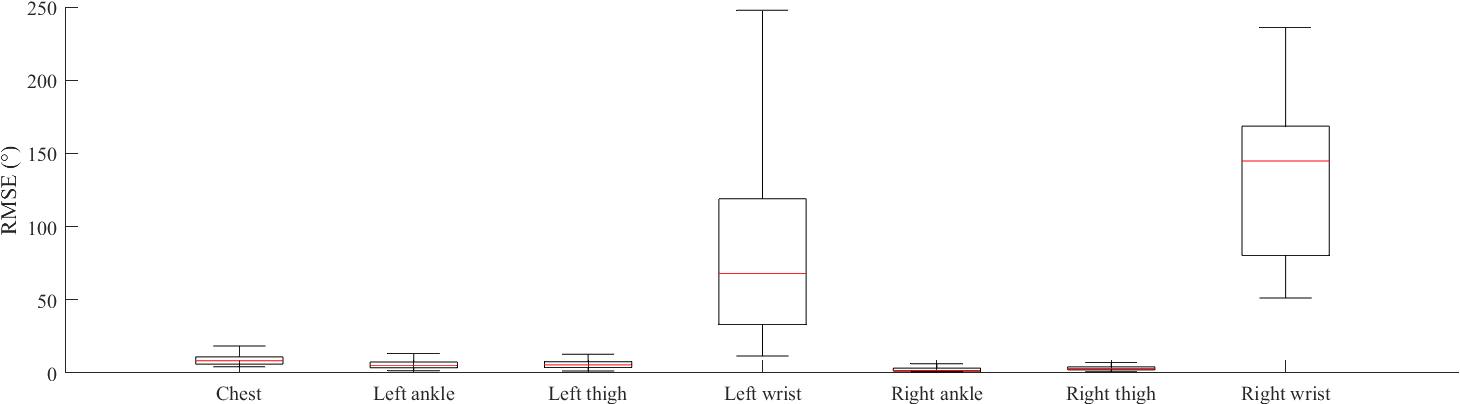


**Figure S6** Good morning yaw angle box plots

**Push-Ups**


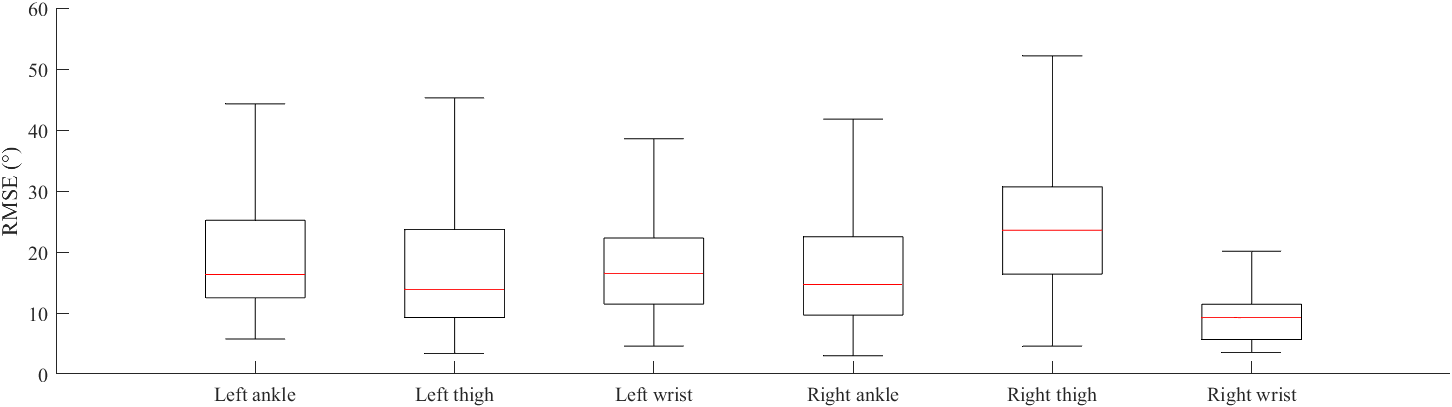


**Figure S7** Push-up pitch angle box plots


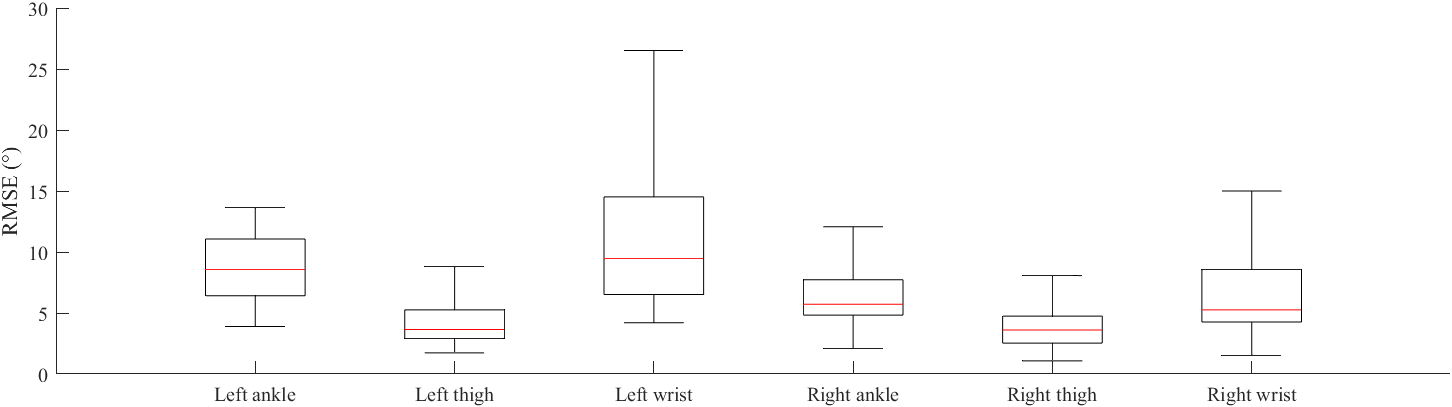


**Figure S8** Push-up roll angle box plots


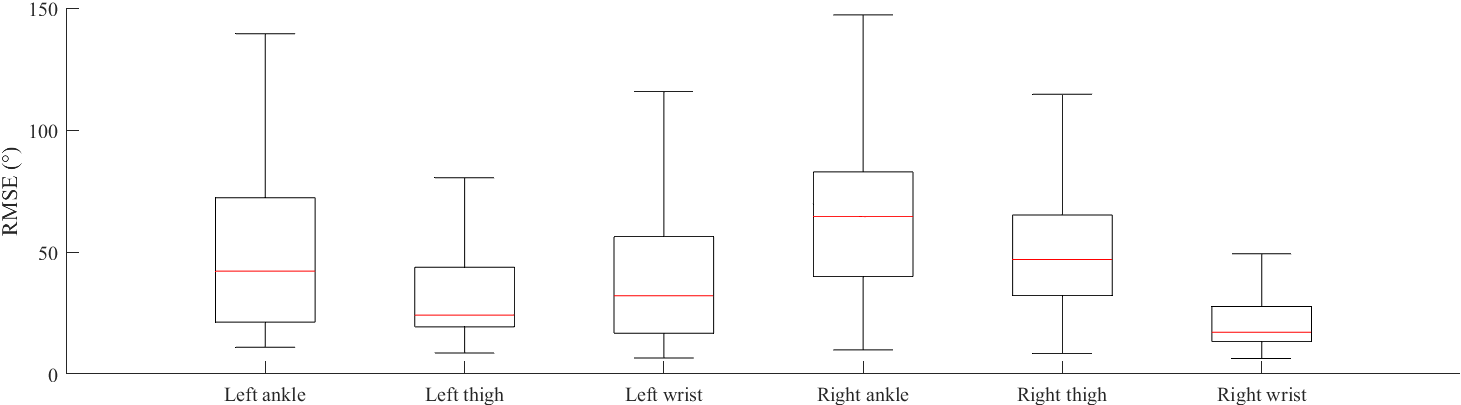


**Figure S9** Push-up yaw angle box plots

**Chair dips**


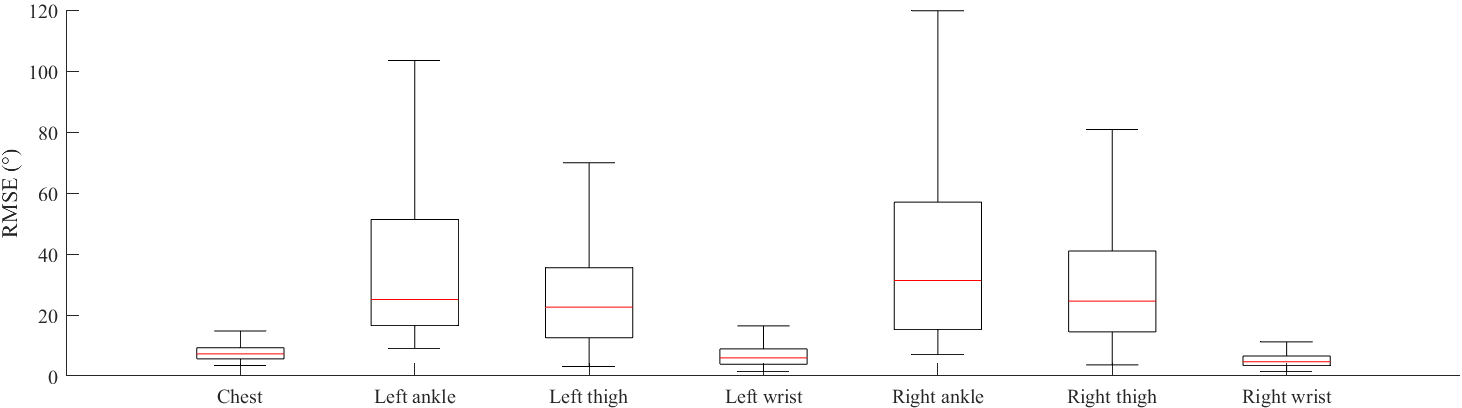


**Figure S10** Chair dip pitch angle box plots


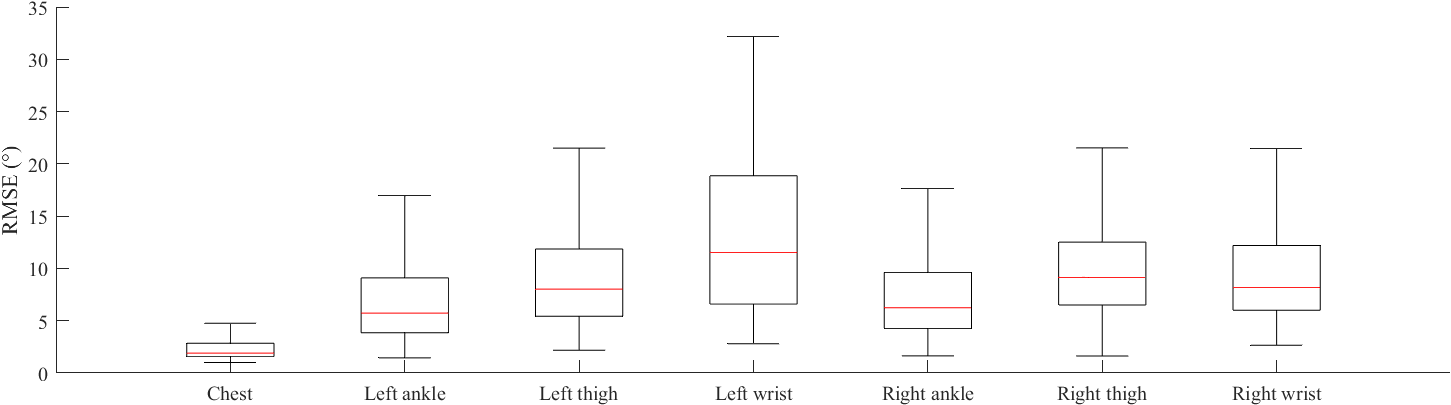


**Figure S11** Chair dip roll angle box plots


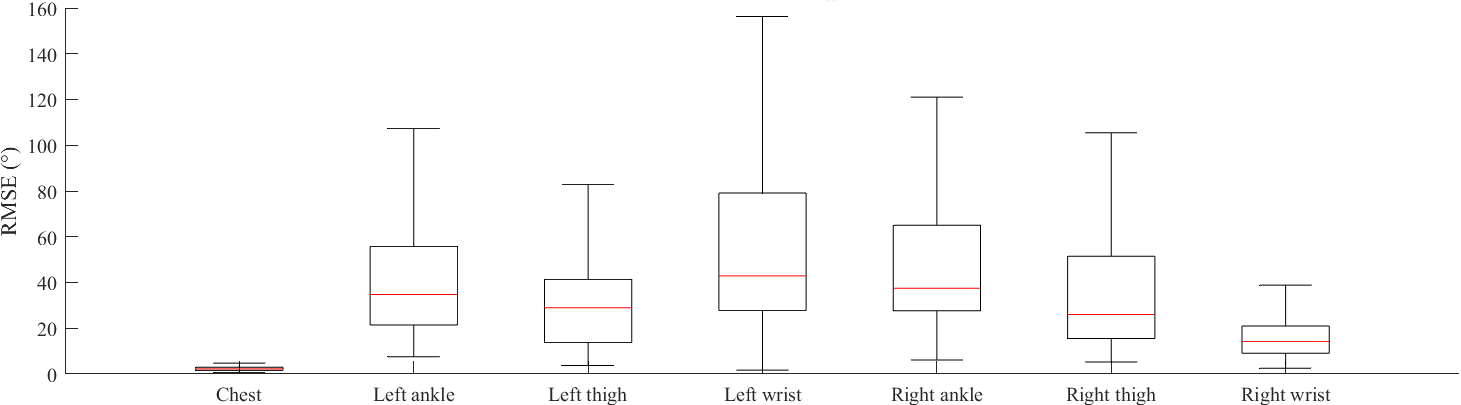


**Figure S12** Chair dip yaw angle box plots
